# About the nature of contextual impairments revealed by FN400 abnormalities in schizophrenia

**DOI:** 10.3389/fnhum.2013.00060

**Published:** 2013-03-01

**Authors:** Fabrice Guillaume, Emmanuel Stip, Guy Tiberghien

**Affiliations:** ^1^Laboratoire de Psychologie Cognitive (CNRS UMR 7290), Aix-Marseille UniversitéMarseille, France; ^2^Centre de Recherche F-Séguin, Hôpital Louis-H LafontaineMontréal, QC, Canada; ^3^Department of Psychologie, Laboratoire Langage, Cerveau, Cognition (L2C2, UMR 5304), Institut Universitaire de FranceBron, France

Face recognition is performed under conditions of ongoing ecological change in our daily lives. In order to recognize someone in such circumstances, it is necessary to ignore contingent perceptual modifications such as clothing, makeup, facial expression, and environmental context. In contemporary research on episodic memory, a distinction is commonly made between familiarity and recollection. Familiarity is generally conceptualized as an automatic, fluent, and effortless memory process, whereas recollection is regarded as more controlled and effortful (Yonelinas, 2002). Familiarity is a convincing memory phenomenon that occurs when someone recognizes an already-seen stimulus without recollecting the contextual details of the prior learning episode (Mandler, 2008). For example, we have all experienced a feeling of familiarity toward a face without remembering when or where we last saw the person, what his/her name is, or certain differences in his/her physical features. Electrophysiological methods (ERP) are well suited to detecting anomalies in the sequence of cognitive processes taking place during recognition. ERPs have proven sensitive to memory retrieval based on both familiarity and recollection, and provide an opportunity for clarifying the behavioral deficits observed in schizophrenia. In recognition-memory experiments that compare ERPs on hits and correct rejections (the so-called ERP “old/new” effects), familiarity has been associated with a mid-frontal N400 effect (FN400), whereas recollection has been associated with a later parietal old/new effect, also called the late positive complex or LPC (Rugg and Curran, 2007). Although familiarity and recollection are undeniably distinct memory experiences, theoretical controversy currently abounds with respect to the neural correlates of familiarity (Mecklinger et al., 2012; Paller et al., 2012). For example, some authors consider that the neurocognitive processes supporting conceptual priming also drive familiarity (Paller et al., 2007). In this view, the FN400 is not different from the N400 that is linked to implicit semantic access during language processing, and more generally during the processing of all meaningful stimuli, including faces (Voss and Federmeier, 2011). But other authors continue to offer evidence that FN400 is functionally distinct from N400 (Bridger et al., 2012).

In a recent study, we proposed that schizophrenia patients present a face-recognition deficit when perceptual changes occur between the study phase and the recognition test. They reject old faces to a greater extent than do comparison subjects whenever the face's background or expression has changed. Interestingly, this deficit is accompanied by suppression of the FN400 old/new effect in patients with schizophrenia, but not in controls, suggesting neurocognitive dysfunctioning related to the mechanisms underlying the emergence, assessment, or utilization of familiarity—as indexed by the FN400 old/new effect (Guillaume et al., 2012a,b).

Based on the possible analogy between FN400 and N400, and in accordance with current neuroanatomical evidence in favor of frontal and temporal sources for these ERP components (Halgren et al., 2002), Amoruso and collaborators recently commented on our results in the present journal (Amoruso et al., 2012). The authors proposed that the FN400 component “might be reflecting a more general process regarding implicit contextual facilitation.” In this view, frontal regions would play a key role in updating ongoing contextual information and integrating it into semantic knowledge about the target-context association stored in the temporal regions. Although we agree with the proposal that the abnormal FN400 modulations found in schizophrenia can be explained in terms of disrupted contextual-cue processing in the frontotemporal network, it is not clear whether the suppression of the FN400 old/new effect can be (1) generalized to any contextual information such as that provided by the semantic or social context, and (2) restricted to implicit processing.

In previous work, we showed that when patients with schizophrenia have to exclude faces on the basis of perceptual changes during exclusion tasks, their performance does not differ from controls (Guillaume et al., 2007). Thus, contextual impairments observed in schizophrenia are not completely independent of the task-processing context and the participants' intentions at retrieval time. The deficit observed in schizophrenia has to do with the ability to ignore the study-test perceptual mismatch in order to recognize faces, and to process the perceptual change separately from the face's identity. Moreover, recent investigations suggest that modulations on FN400 are not restricted to the implicit processing of context. In a face-recognition study, we showed recently that the observed FN400 modulations did not depend solely on the implicit processing of the context but also on the retrieval orientation taken with respect to the task-processing context (Guillaume and Tiberghien, 2013). Similar retrieval orientation effects on FN400 have been found in a reality-monitoring study (Rosburg et al., 2013). These findings clearly suggest that the neurocognitive mechanisms that modulate the FN400 can be influenced by conscious access during recognition.

The suppression of the FN400 old/new effect observed in schizophrenia under perceptual-change conditions may therefore reflect an abnormal retrieval mode, which in turn leads to a deficit in the processing of contextual information. Everything points to the conclusion that schizophrenia patients base their recognition on a global strategy that matches perceptual features within a conjunctive representation. This enables them to process face identity and perceptual changes separately, and leads to the suppression of both the feeling of familiarity and the FN400 old/new effect. On the other hand, the recognition deficit observed in our studies concerns the retrieval of intra-item perceptual features and cannot be generalized to all associative retrieval processes requiring inter-item or inter-domain binding, as in the case of conceptual or semantic priming. In this view, the abnormal FN400 modulations found in schizophrenia would reflect a very conservative encoding specificity in the perceptual domain rather than a general deficit in the processing of implicit contextual information. This proposal converges with previous neurotransmission studies showing an “analogical processing excess” in schizophrenia (Tassin, 1996). Further research is needed to determine whether FN400 suppression in mismatch conditions is observed with more conceptual materials such as words or face-occupation associations. If this turns out to be true, and only in this case, it would confirm the idea that the abnormal FN400 related to a general deficit in implicit contextual processing during semantic integration in schizophrenia.
